# Intact cutaneous C fibre afferent properties in mechanical and cold neuropathic allodynia

**DOI:** 10.1016/j.ejpain.2009.10.001

**Published:** 2010-07

**Authors:** Richard Hulse, David Wynick, Lucy F. Donaldson

**Affiliations:** Department of Physiology and Pharmacology, University of Bristol, Bristol, UK

**Keywords:** Neuropathic pain, Ongoing activity, Primary afferent, C fibre, A fibre

## Abstract

Patients with neuropathy, report changes in sensory perception, particularly mechanical and thermal allodynia, and spontaneous pain. Similar sensory changes are seen in experimental neuropathies, in which alteration in primary afferent properties can also be determined. The neural correlate of spontaneous pain is ongoing activity in sensory afferents. Mechanical and heat allodynia are thought to result from lowered activation thresholds in primary afferent and/or central neurones, but the mechanisms underlying cold allodynia are very poorly understood.

We investigated nociceptive behaviours and the properties of C and A fibre intact afferents running adjacent to damaged afferents following a partial ligation injury of the saphenous nerve (PSNI). Animals developed mechanical and cold allodynia by 3 days after PSNI. Intact mechanosensitive C fibre afferents developed ongoing activity, and had slower conduction velocities 3 and 7 days following nerve injury, with no change in mechanical threshold. There was a large increase (∼46-fold) in calculated afferent input 3 days after nerve injury, as a result of the ongoing activity in these fibres. Mechano-cooling-sensitive C fibre afferents showed both enhanced cooling-evoked firing, and increased ongoing activity. The afferent barrage associated with mechano-cooling-sensitive afferents was increased 26-fold 7 days after nerve injury. We observed no differences in the properties of intact A fibre mechanosensitive afferents.

These studies demonstrate for the first time that the altered nociception seen after PSNI is associated with ongoing activity and enhanced cooling-evoked activity in intact C fibre afferents in the saphenous nerve, with no concurrent alteration in A fibre afferents.

## 1 Introduction

Mechanical and thermal allodynia are changes in sensory perception that impact profoundly on patients with neuropathy (Gracely et al., 1992, Jorum et al., 2003). In neuropathy, alterations in sensory perception, and behaviour in rodent models such as reduced withdrawal thresholds, have been linked to ongoing changes in the properties of peripheral and central sensory neurones (Gracely et al., 1992). Central sensitisation is induced by increased C fibre nociceptor barrage into the dorsal horn (Millan, 1999), hence the occurrence of ongoing activity in primary afferents has been widely studied.

Changes in C fibre ongoing activity have been reported in relatively few peripheral nerve injury studies (Wu et al., 2001, Shim et al., 2005, Djouhri et al., 2006, Roza et al., 2006, Ji et al., 2007). In contrast, many studies have shown that myelinated A fibre afferents develop ongoing activity after axotomy (Tal et al., 1999, Michaelis et al., 2000, Ritter et al., 2007), constriction injury (Kajander and Bennett, 1992), or spinal nerve ligation (SNL) injury (Liu et al., 2000, Ma et al., 2003, Ji et al., 2007), with few concurrent changes in C fibre afferents. Following spinal or peripheral nerve section, the onset of A fibre ongoing activity correlates well with the onset of mechanical allodynia (Liu et al., 2000). Ongoing activity occurs primarily in damaged A fibre afferents (Sapunar et al., 2005), but may also occur in uninjured adjacent fibres (Michaelis et al., 2000, Ma et al., 2003, Ji et al., 2007). The level of ongoing activity in uninjured C fibre primary afferents adjacent to injured fibres is highly correlated with spontaneous foot-lifting (SFL) behaviour after both spinal nerve axotomy/ligation (Djouhri et al., 2006). It is hypothesised that activity in the intact afferents running adjacent to an injured afferents may be fundamental in the generation of altered nociception in nerve injury (Wu et al., 2001).

Following nerve injury, central sensitisation is initiated, and possibly maintained by sustained alterations in primary afferent properties (Millan, 1999). The central changes in dorsal horn neurones, including lowered thresholds, induced by ongoing activity in nociceptors, are thought to account for behavioural changes following nerve injury (Millan, 1999, Woolf, 2004). Reduction in primary afferent mechanical threshold was not thought to occur in cutaneous primary afferents (Andrew and Greenspan, 1999, Koltzenburg et al., 1999) but several recent reports lend support to the hypothesis that such primary afferent sensitisation contributes to both neuropathic (Shim et al., 2005, Chen and Levine, 2007) and inflammatory mechanical allodynia (Dunham et al., 2008). The neuronal mechanisms of cold allodynia are poorly understood, but evidence suggests properties are altered in both cooling and noxious cold-responsive primary afferents following peripheral nerve injury (Takahashi et al., 2003, Ji et al., 2007).

In this study we have characterised the properties of mechano-cooling-sensitive primary afferent nociceptors with intact peripheral receptive fields, in a recently described model of partial sensory nerve injury (Walczak and Beaulieu, 2006), to define changes in mechanical or cold thresholds, and relate these to the properties of the intact A and C primary afferents.

## 2 Methods

A total of 53 male Wistar rats (250–350 g) were used in these studies. Animals were housed under standard 12:12 h light:dark cycles and had access to standard chow and water ad libitum. All experiments were carried out in accordance with the United Kingdom Animals (Scientific Procedures) Act 1986 and University of Bristol Ethical Review Panel guidelines.

### 2.1 Nerve injury model

Partial saphenous nerve injury (PSNI) was carried out as previously described (Walczak and Beaulieu, 2006) on 26 rats. Briefly, rats were anaesthetised with 4% halothane. Animals were maintained areflexive under deep surgical anaesthesia throughout the procedure, with 2% halothane in oxygen. The saphenous nerve was exposed through an incision in the inguinal fossa of the right hind limb, to expose the femoral vasculature with the saphenous nerve running alongside. The saphenous nerve was isolated from the surrounding tissues, and the nerve trunk gently split approximately in half longitudinally using watchmakers forceps. One half of the split nerve trunk was then tightly ligated using 4.0 sterile silk suture. The overlying connective tissue and skin were sutured with a 4.0 sterile silk suture and the animals were allowed to recover.

Control animals for nerve injury were both sham operated (*n* = 4) and naı¨ve (*n* = 4) for the behavioural experiments, and only naı¨ve controls for the electrophysiological study as sham operated animals showed no significant behavioural changes when compared to naı¨ve controls.

### 2.2 Nociceptive behavioural tests

Sixteen rats (intact *n* =  4, sham *n* = 4 and NI *n* = 8) were placed in transparent Perspex enclosures and habituated to the environment the day before behavioural testing, and for at least 15 min until they settled, before each testing session. Behavioural testing consisted of the following: observation of spontaneous foot-lifting (lifting of the hind paw not associated with any obvious stimulus e.g walking) for 5 min; measurement of mechanical withdrawal threshold, and response to skin cooling evoked by the application of acetone onto the plantar surface of the hind paw. All tests were applied to both hind paws. Behavioural tests were performed 6 and 4 days prior to surgery, then every 2–3 days thereafter until 14 days after nerve injury.

Mechanical withdrawal thresholds were determined using a series of calibrated von Frey filaments (Linton Instruments, UK) ranging from 4 g to 100 g or until 100% withdrawal was achieved from the five applications. Each filament was applied to the medial plantar surface of each hindpaw five times for a maximum period of 5 s. Mechanical withdrawal thresholds, as the force at which the animal withdrew each paw 50% of the time, were then calculated from the stimulus;response curves generated.

Cooling responses were determined from the response to a single drop of acetone applied to the plantar surface of the hindpaw that was applied a total of three times. Cumulative responses to cooling were scored as flinching or paw shaking equalling a score of 1 and no response scoring 0. Each testing session therefore generated a total score of between 0 and 3. Development of cold allodynia over the period of testing (20 days total, 14 days after injury) was calculated by plotting flinching/shaking scores for each animal against time. Mean AUC for 0–3, 5–8, and 10–14 days was calculated for each group of animals. Behavioural testing was performed by a single operator (RH) blinded to animal treatment.

### 2.3 Gait analysis

A cage was divided into a 3 × 3 grid by tape on the floor of the cage. Rats were placed, one at a time, into the middle square of the grid, and the number of line crossings, rearings (time spent on hindpaws) and duration of total rearings were recorded for a 5 min period.

Rats were habituated to walking along a 1 m long narrow platform covered in paper, with the housing cage at the opposing end to encourage movement in one direction. Hind paws were then coated in yellow paint and the rats were allowed to walk from the same point to the home cage along the platform, three times. The time taken, the number of strides in the track, the distance between strides and the contact of the hindpaws with the supporting surface were measured.

### 2.4 Neurophysiological characterisation of primary afferents

Neurophysiology was performed on three groups of animals, those that had had PSNI 3 and 7 days previously (day 3, *n* = 10, day 7, *n* = 8; total 18) and uninjured animals (*n* = 19).

Animals were anaesthetised (60 mg/kg i.p.) and maintained deeply anaesthetised and areflexive on sodium pentobarbital (20 mg/kg/h i.v.). The trachea was cannulated to maintain the airway and the external jugular vein and an artery (femoral) cannulated for anaesthetic administration, blood pressure monitoring and/or drug delivery. Body temperature was maintained within physiological limits by means of a feedback controlled heater and rectal thermister. The ambient temperature was carefully controlled in the laboratory during all the experiments, and did not vary between experiments on PSNI or control animals, to ensure that cooling responses would not be attributable to changes in skin temperature due to altered environment. At the end of all experiments, rats were killed by an overdose of sodium pentobarbital.

The right saphenous nerve was exposed mid-thigh via an incision from the inguinal fossa to a point just distal to the knee joint and was isolated from the surrounding tissue. A pool of warmed paraffin oil was made of the surrounding skin to prevent dehydration and, following removal of the epineurium, fine filaments of the saphenous nerve were teased to enable differential recording of neuronal activity via bipolar platinum wire electrodes placed proximal to the PSNI ligation. Filaments were teased until they contained a small number (often ⩽2 but occasionally 3) identifiable afferents, as determined by individual waveform analysis using C.E.D. Spike 2 v5 (Cambridge Electronic Design, Cambridge, UK), and stimulation of individual receptive fields when determining CV. Multiple filaments were studied in each animal in order to reduce the number of animals used, in line with UK legislation on reduction of animal numbers (the UK Animals (Scientific Procedures) Act, 1986). When collecting data from several filaments in the same animal, only units with non-overlapping receptive fields were characterised, so as to avoid sensitisation by previously applied stimuli. Action potentials were amplified and passed through a C.E.D. 1401 analogue to digital converter. Spikes were recorded and analysed using C.E.D. Spike 2 v5 software. In filaments where >1 unit was recorded, each individual unit was isolated, and activity sorted online using waveform analysis in Spike 2.

In all animals, irrespective of treatment group, the search stimuli and identification of afferents for study was performed in the same manner. The receptive fields (RF’s) of primary afferent fibres (PAF’s) were initially identified by a mechanical search stimulus, first by a gentle brush on the skin in the innervation territory of the saphenous nerve and then using gentle pinch with blunt forceps to probe the area. Conduction velocities (CV) of identified units were then determined using constant current monopolar electrical stimulation (up to 100 V, 0.5 ms duration) (Kress et al., 1992) of the receptive field. C and A fibre conduction velocity boundaries were <$1\;\text{m}\;{\text{s}}^{-1}\;(\text{C})\text{,}\;2\text{"–"}10\;\text{m}\;{\text{s}}^{-1}\;(\text{A}\delta )$ and >$10\;\text{m}\;{\text{s}}^{-1}\;(\text{A}\beta )$. These boundaries were determined from saphenous nerve compound action potentials (CAP) recorded in the same region of the saphenous nerve, performed in animals of the same sex and weight as those used for subsequent experiments (Dunham et al., 2008). 6/79 units classified as C fibres had CV >$1\;\text{m}\;{\text{s}}^{-1}$, and all of these units had CV <$1.2\;\text{m}\;{\text{s}}^{-1}$. These were classified as C fibres on a combination of CV and the shape of the action potential (Gee et al., 1999). Following the identification of PAF RF and CV calculation, and after a resting period of at least 2 min, ongoing activity was recorded for a continuous 100 s period with no stimulation of the receptive field. Ongoing activity rate was measured prior to any further characterisation of the unit, to ensure that repeated stimulation of the receptive field did not affect this measure. Mechanical thresholds of the afferent neurones (units) were determined using calibrated von Frey hairs (Linton Instruments, Norfolk, UK) as reported in previous studies using both *in vivo* and *in vitro* preparations (Kumazawa and Perl, 1977, Lynn and Carpenter, 1982, Leem et al., 1993, Koltzenburg et al., 1999, Chen et al., 2007, Chen and Levine, 2007, Dunham et al., 2008). Filaments were applied to the most sensitive region of the receptive field for approximately 5 s. The weight of the lowest force filament that reproducibly evoked activity (>3 action potentials) was defined as the threshold of the unit, as previously described using a similar method (Dina et al., 2004). It should be noted that hand-held von Frey hairs give an approximation of the mechanical thresholds of primary afferent units as application of a range of hairs exerts incremental, discrete forces rather than a continuous force on the receptive field. Units that did not respond to stimuli >180 g were not included in the analysis as it could not be determined whether these units were “silent” nociceptors (Michaelis et al., 1996) or whether the receptive field could not be located.

One drop (∼50 μl) acetone was then applied to the RF and evoked activity was recorded. This stimulus results in a drop in subcutaneous temperature of ∼5 °C in the rat (Hulse, R., Dunham, J., unpublished observations) and ∼10 °C in mouse (Colburn et al., 2007), and thus represents a non-noxious stimulus. The criteria for a cold response in these CMC mechanoreceptors were a brief increase in firing rate above any previous ongoing firing, during the application of the acetone, and a cessation in firing on re-warming of the skin (Iggo, 1960). Classical cooling sensitive units (Hensel and Zotterman, 1951, Hensel, 1981) were not included in the study. These units could be easily identified as they usually have bursting pattern, ongoing activity, at the skin temperatures in the animals used in this study (∼25 °C), and they are not mechanically sensitive (Hensel et al., 1960). The C fibre units in the study were all mechanoreceptors (CM), some of which also responded to acetone induced skin cooling (CMC), which have been previously classified as mechanoreceptors (Hensel et al., 1960, Iggo, 1960). Responses to cutaneous warming were not tested in these experiments.

Ventroflexion of the hindpaw has been described in models of nerve injury involving motor and sensory damage, and in this model in the mouse (Hulse et al., 2008) but was not previously noted in this model in the rat (Walczak et al., 2005). Ventroflexion was evident in awake animals when no weight was borne on the paw. To assess the degree of ventroflexion, foot length was recorded by measuring the length from heel to middle toe (mm) of both ipsilateral and contralateral hindpaws under general anaesthesia at the beginning of the terminal experiments.

### 2.5 Afferent selection criteria and potential bias

In both control and PSNI animals, afferents were selected for study on their response to a mechanical search stimulus (brush, pinch with forceps, high intensity pressure with a blunt glass probe). The sample was therefore biased to mechanically-sensitive afferents. Mechanical search stimuli excluded mechanically insensitive cooling afferents. There was some selection bias towards C fibre afferents but A fibre afferents that responded to mechanical stimuli, in either group of animals, were also included in the analysis.

### 2.6 Statistical analysis

All data shown are means ± SEM unless otherwise noted in the text or figure legends. Data were tested for normality when possible, and appropriate versions of tests (parametric or non-parametric 1- or 2-way ANOVA, and two group tests) were used depending on the normality of the data and the sample size. Behavioural measures (gait and nociceptive testing) were analysed using 2-way ANOVA with appropriate post hoc tests for individual comparisons. One-way ANOVAs with appropriate post hoc tests (noted in the figure legends) were used for other comparisons of three or more groups (ongoing firing rates, thresholds, evoked activity). Comparisons of proportions of afferents with different properties were made using Chi squared test. Hind paw lengths were compared using paired *t*-tests. All statistical calculations were performed in Graphpad Prism 4.00 for Macintosh (GraphPad Software, San Diego California USA, www.graphpad.com). Null hypotheses were rejected if *p* < 0.05.

## 3 Results

### 3.1 Behavioural changes after partial saphenous nerve injury in the rat

**Fig. 1:**
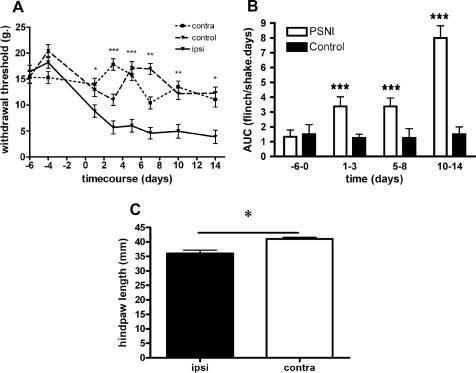
Behavioural changes following PSNI in the rat. (A) Ipsilateral mechanical allodynia was evident after 1 day and reached a maximum by 3 days after PSNI, as there was no statistically significant reduction in threshold from day 3 to day 14. The contralateral limb showed no allodynia compared to the uninjured side, or control rats (2-way ANOVA). (B) Cold flinching/shaking behaviour was significantly increased in paws ipsilateral, but not contralateral to PSNI over the first 3 days, 6–8 and 10–14 days after PSNI. (ANOVA + Bonferroni). (C) Significant ventroflexion of the hindpaw was evident 3 days after PSNI (paired t-test). ^∗^*p* < 0.05, ^∗∗^*p* < 0.01, ^∗∗∗^*p* < 0.001.

As previously reported (Walczak et al., 2005), partial saphenous nerve injury (PSNI) resulted in the rapid onset of ipsilateral mechanical allodynia, with mechanical threshold being significantly reduced one day after injury (thresholds Day −6: 16.4 ± 1.2 g; Day +1: 8.8 ± 1.1 g, *p* < 0.05). Maximum allodynia was achieved 3 days after injury, and did not resolve over the next 11 days (Fig. 1A). Neither sham operated nor naı¨ve control animals displayed mechanical allodynia or significant responsiveness to acetone.

Groups (untreated, injured side, and contralateral side) were compared by calculation of the areas under the curves (AUC) in the control period (Days −6 to 0), the first 3 days, days 6–8, and days 10–14 to determine changes in cooling-evoked behaviour at the times when mechanical allodynia was fully developed, and maintained. No significant differences in cooling-evoked behaviours were evident prior to nerve injury. Cooling evoked behaviour after PSNI was increased ipsilaterally in the first 3 days and in the periods 6–8 and 10–14 days after PSNI (Fig. 1B).

PSNI animals showed no spontaneous foot-lifting behaviour at any time after surgery, nor were autotomy, or alteration in stride length or width noted (not shown). Marked ventroflexion was obvious in the anaesthetised animals resulting in a significant reduction in heel-middle toe length in the nerve-injured paw, compared to the contralateral paw, 3 (Fig. 1C) and 7 days after injury (not shown).

### 3.2 Electrophysiological properties of primary afferent neurones with intact peripheral receptive fields after PSNI

Afferents were separated into and analysed as groups of C or A fibre neurones based on conduction velocities measured from compound action potentials recorded in the same preparation in rats of similar age and weight (Dunham et al., 2008). A total of 79 C fibre mechanically sensitive units were studied in control rats, 33 units in day 3 and 30 units in day 7 PSNI rats, of which 59, 30 and 22 were C-mechanoreceptor (CM) fibres respectively. The other units responded to both mechanical and cooling (acetone) stimulation (CMC units).

**Fig. 2:**
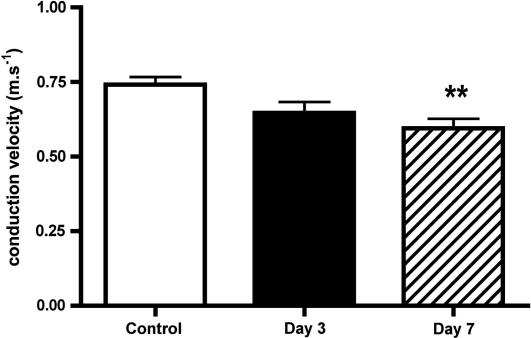
Conduction velocities were significantly reduced in intact C fibre afferents 7 days after PSNI (Kruskal-Wallis + Dunn’s) ^∗∗^*p* < 0.01.

Conduction velocities of the CM afferents were in the range reported for rat saphenous nerve (Iggo, 1960). CVs were decreased after PSNI and were significantly slower than controls after 7 days (Fig. 2). There was no change in A fibre conduction velocities after nerve injury.

#### 3.2.1 Ongoing activity in mechanically sensitive primary afferent fibres

**Fig. 3:**
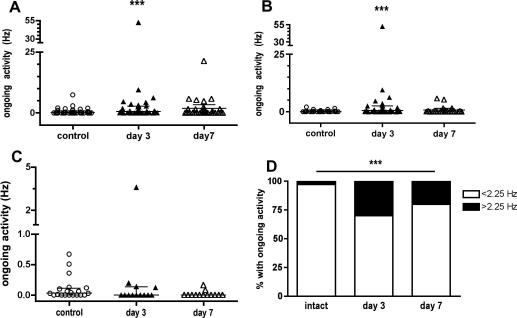
(A) Ongoing activity in all mechanically-sensitive C fibre afferents was significantly increased 3 days after PSNI (^∗∗^*p* < 0.001, Kruskal-Wallis + Dunn’s). At 7 days ongoing activity was evident but was not significantly different from either day 3 or control. Bars indicate median ± IQR. (B) Ongoing activity in mechanically-sensitive C fibre afferents excluding CMC units was similarly significantly increased 3 but not 7 days after PSNI (^∗∗^*p* < 0.001, Kruskal-Wallis + Dunn’s). (C) Ongoing activity in A fibre afferents was not significantly changed by PSNI. Bars indicate median ± IQR. (D) The proportions of C fibres with ongoing activity >2.25 Hz was significantly increased at both 3 and 7 days after PSNI (Chi squared, see also Table 1).

Cutaneous C fibre mechanoreceptor afferents in control animals showed a very low level of ongoing activity, with a mean level of ongoing activity of 0.33 ± 0.11 Hz (median ± median absolute deviation (MAD = IQR/2), 0.06 ± 0.08 Hz). In contrast the mean level of ongoing activity 3 days after PSNI was 3.02 ± 0.6Hz (0.56 ± 1.2 Hz, *p* < 0.001, Fig. 3A), and this approached control levels again at 7 days, when the firing rate was 1.8 ± 0.75 Hz (0.14 ± 0.77 Hz). When only the mechanically sensitive units not including the CMC were analysed, there was a significant increase in median ongoing firing rate at 3, but not at 7 days after PSNI (Fig. 3B). In contrast, no difference in ongoing activity of A fibres with intact peripheral receptive fields was noted after nerve injury (Fig. 3C).

In order to compare the proportions of mechanically-sensitive afferents with ongoing activity, we determined a rate of firing to define as ongoing activity. The rate of firing that is defined in the literature as resting/spontaneous/ongoing activity is very variable. Values range from very low (1action potential in 5 min (Ali et al., 1999, Wu et al., 2001), 0.1 Hz (Shim et al., 2005)), to >3 Hz (Ritter et al., 2007); others do not define a specific frequency of firing (Ma et al., 2003). In nerve injury models where ongoing activity correlated strongly with SFL, the mean firing frequency of C fibre neurones was approximately 1 Hz (Djouhri et al., 2006).

We therefore compared the proportions of afferents in control, days 3 and 7 groups with either 0.1 Hz (Shim et al., 2005), the mean value from (Djouhri et al., 2006) of 1 Hz, and 2.25 Hz ongoing activity as cut-offs to define the presence of ongoing activity. The 2.25 Hz value is derived from the mean control (naı¨ve) ongoing firing rate +2 × SD (0.96 Hz) in our control animals, as although ongoing firing was not normally distributed, the mean firing rate has been used to calculate afferent barrage (Djouhri et al., 2006). Irrespective of the value used, there was a significant change in the proportions of afferents showing ongoing firing (Table S1. See the online version at doi:10.1016/j.ejpain.2009.10.001). We used the proportions generated by the cut-off of 2.25 Hz to calculate the total afferent input to the spinal cord, as this would be expected to result in an underestimate, rather than overestimate of the total afferent input resulting from PSNI.

**Table S1:** Proportions of CM fibres showing ongoing activity after PSNI.

	Ongoing activity rate					
	<0.1 Hz	>0.1 Hz	<1 Hz	>1 Hz	<2.25 Hz	>2.25 Hz
Control	47	32	71	8	77	2
Day 3	11	22	22	11	23	10
Day 7	14	16	20	10	24	6

*p*	0.03		0.003		<0.0001	

Proportions of fibres showing different rates of ongoing firing are shown for three different firing rates, based on literature values (0.1 Hz, (Shim et al., 2005), 1 Hz (Djouhri et al., 2006)) and values in this study (2.25 Hz, detailed in the text). Proportions of fibres with ongoing activity were significantly increased irrespective of the rate used to define ongoing activity (Chi squared).

Only 2/79 (3%) control C fibre mechanically-responsive afferents exhibited ongoing activity >2.25 Hz. Three and seven days after PSNI, 10/33 (30%) and 6/30 (20%) C fibres exhibited ongoing activity >2.25 Hz. At both times this represented a significantly greater proportion of C fibre afferents with ongoing activity than in controls (*p* < 0.001, Fig. 3D). Of the fibres with ongoing activity at day 7, 4 of the 6 with activity > than 2.25 Hz were CMC units. These fibres were mechanically sensitive, and their ongoing activity would contribute to sensitisation of mechanosensitive spinal neurones, so they were included in the analysis.

**Fig. S1:**
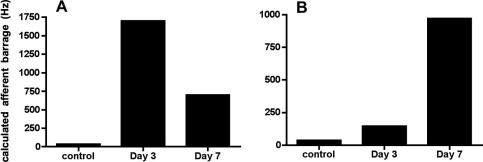
Calculated afferent barrage reaching the spinal cord following PSNI. (A) Barrage from all mechanosensitive C fibres was increased 46-fold 3 days after, and 18-fold 7 days after PSNI. (B) Barrage from mechano-cooling-sensitive (CMC) afferents was slightly increased (∼4-fold) 3 days, but greatly increased (26-fold) 7 days after PSNI.

In order to induce central sensitisation to mechanical stimulation, a significant C fibre barrage, presumably in C fibre mechanoreceptors, is required. For example, in order to induce spinal neuronal wind-up, (an experimental correlate of central sensitisation), electrical stimulation at C fibre intensity and frequencies from as low as 0.5 Hz (Grubb, 1998) is often used. In order to determine the approximate afferent barrage reaching the spinal cord as a result of the altered properties of these C fibre afferents after PSNI, we estimated the afferent input under control and PSNI conditions. These calculations are based on: (1) the number of unmyelinated sensory afferents in the rat saphenous nerve (3750, (Baron et al., 1988), (2) the assumption that PSNI leaves approximately 50% of these with an intact RF, (3) the proportions of afferents in each condition with ongoing activity >2.25 Hz, and (4) the mean firing rates of the afferents with ongoing activity in each group, in a manner similar to that calculated for the mSNI model (Djouhri et al., 2006). These calculations show that the total estimated C fibre barrage reaching the CNS following PSNI increased from 37 Hz in control animals to 1.7 KHz (∼46-fold) at day 3, and 0.7 KHz (∼18-fold) at day 7 (Fig. S1. See the online version at doi:10.1016/j.ejpain.2009.10.001).

#### 3.2.2 Mechanical thresholds

After nerve injury we did not attempt to classify afferents by threshold, as we hypothesised that there would be a reduced mechanical threshold in intact afferents adjacent to injured afferents. Overall there was no significant difference in median mechanical threshold in day 3 (10 ± 13 g, median ± MAD) or day 7 (4 ± 13 g) PSNI rats compared to control (10 ± 11 g) (not shown). The median threshold tended to be lower 7 days after PSNI, suggesting that a proportion of afferents may show lowered thresholds, as we have previously shown in inflammation (Dunham et al., 2008). We hypothesised that ongoing activity might indicate afferents that had been sensitised by the injury, which might have lower thresholds. Thresholds were therefore compared in fibres with and without ongoing activity at days 3 and 7. There were no significant differences in mechanical thresholds in these fibres, although again there was a trend for thresholds to be lower in spontaneously active fibres at day 7 (*p* = 0.07, not shown). A fibre afferents also showed no change in mechanical threshold on either day 3 or day 7 after PSNI (not shown).

#### 3.2.3 Ongoing and evoked activity in cooling-responsive afferents

**Fig. 4:**
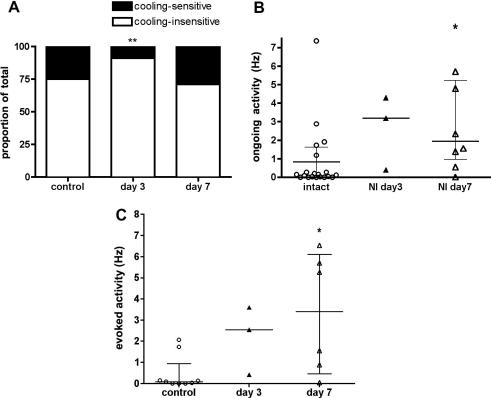
Mechano-cooling-sensitive afferent properties after PSNI. (A) The proportions of CMC afferents that showed a response to acetone-evoked cooling were significantly decreased at day 3 PSNI compared to either controls or day 7. (Chi squared, *p* = 0.011). (B) The amount of ongoing activity exhibited by C fibre cooling afferents increased with time after PSNI and was significantly higher than control at day 7 (^∗^*p* < 0.05, Kruskall-Wallis + Dunn’s). Bars represent median ± IQR (day 3 median). (C) The amount of activity evoked by acetone stimulation increased over time, and was significantly increased 7 days post-PSNI compared with control (^∗^*p* < 0.05, Kruskall-Wallis + Dunn’s). Bars represent median ± IQR.

Twenty five percent (20) of the 79 mechanically-sensitive C fibres also responded to an acetone cooling stimulus in control animals. No A fibre afferents studied had a cooling response. The proportion of the total number of CMC afferents significantly decreased 3 days after PSNI to 9% (3/33), (*p* = 0.011) but by day 7 PSNI CMC unit proportions had returned to control levels (27%, (8/30) Fig. 4A). Unlike the all mechanically-sensitive C fibres, in which ongoing activity was significantly increased at day 3, in just CMC fibres, ongoing activity was significantly increased at 7 days post-PSNI (Fig. 4A). The proportions of C fibre cooling-sensitive afferents with ongoing activity >2 SD from the mean control activity were 1.25% in control animals, 3% at day 3 and 11% at day 7 post-PSNI. The control ongoing activity in cooling-sensitive afferents was 0.82 ± 0.4 Hz (0.14 ± 0.37 Hz median ± MAD), and this increased to 2.6 ± 1.2 Hz (3.2 Hz (4 Hz range) and 4.7 ± 2.5Hz (1.9 ± 2.6 Hz) at days 3 and 7 respectively (*p* < 0.05, Fig. 4B). In addition to this, the amount of activity evoked by the acetone stimulus applied to the skin was also significantly increased in these afferents at day 7 post-PSNI (Fig. 4C, *p* < 0.05). Thus CMC afferents became significantly more responsive to skin cooling post-PSNI, in addition to developing ongoing activity.

The afferent barrage attributable to just the mechano-cool fibres was calculated as with that associated with all mechanically-sensitive afferents, as it is hypothesised that activity in these afferents could contribute to cold sensitisation. In contrast to the total afferent input from mechanosensitive afferents, but in keeping with the increases in their ongoing activity, the total cooling-afferent-associated barrage was increased slightly (∼4-fold) at day 3 but was greatly increased at day 7 (∼26-fold) (Fig. S1).

## 4 Discussion

Partial saphenous nerve injury results in the rapid development of behavioural mechanical and cold allodynia in the rat (these data (Walczak et al., 2005)), with little effect on gross measures of gait or behaviour, such as exploratory behaviour, or stride length. We did not observe spontaneous foot-lifting in these animals, a behaviour thought to be a robust measure of spontaneous pain (Djouhri et al., 2006 and references therein). We did, however, observe significant ventroflexion of the hindpaw of the damaged hindpaw, which we also observed following mouse PSNI (Hulse et al., 2008).

The C and A fibre afferents studied were all intact, with identifiable, peripheral receptive fields. Although intact, these afferents are not “normal”, as they are likely to be affected by the neuro-inflammation associated with the adjacent injury. PSNI resulted in a significant slowing of the conduction velocities of the intact C fibre afferents, as has been reported in ethanol-induced neuropathy (Chen and Levine, 2007). Slowing of conduction velocity has been described in myelinated afferents in different human neuropathies, including diabetic neuropathy (Bertora et al., 1998, Chen, 1998, Emeryk-Szajewska et al., 1998, Quasthoff, 1998, Ooi and Srinivasan, 2004), where it is usually associated with fibre regeneration (Shefner et al., 1991). It is rarely reported in C fibres (for example (Ali et al., 1999, Ji et al., 2007)), and in PSNI is unlikely to be associated with regeneration. Slowing of nerve conduction velocity is indicative of a change in the excitability of the afferent, representative of a change in ionic conductances following nerve injury (Cherian et al., 1996, Chen and Levine, 2007), but may be associated with alteration of expression level and/or site of expression of sodium, potassium and/or calcium channels, thus altering excitability of the sensory fibre (Quasthoff, 1998, Cummins et al., 2007, Mert, 2007).

Spontaneous foot-lifting has been considered to be a sign of ongoing, or spontaneous pain, that is pain without an obvious stimulus. It has recently been shown to be related to the level of resting (ongoing, spontaneous) discharge in C fibre primary afferents, again a physiological change with no clear evoking stimulus. Although we did not observe SFL in the presence of a dramatic increase in resting C fibre discharge, paw ventroflexion was evident, and we hypothesised that this difference may be due to the magnitude of the afferent barrage generated.

Significant ongoing activity was seen in intact C, but not A fibre mechanosensitive afferents, at 3, but not 7, days after PSNI. The mean ongoing firing rate after PSNI was higher than that reported following spinal nerve ligation (Ali et al., 1999, Wu et al., 2001, Djouhri et al., 2006, Ji et al., 2007); the reasons for this difference are unclear. Although the ongoing activity rate was not increased at day 7, there was an increased number of afferents exhibiting such discharge to approximately 20%, associated with a dramatic increase in the total afferent input to the spinal cord. The net result of the increased firing rate, and the increased proportions of afferents with ongoing activity was that there was a 46, and a 19-fold increase in afferent input to the spinal cord 3 and 7 days respectively after nerve injury. This translates to an approximate afferent input of 1.7 KHz and 0.7 KHz at 3 and 7 days post-PSNI, values less than those in which SFL was apparent (Djouhri et al., 2006). Spontaneous foot-lifting is not always observed in experimental models of nerve injury, and in these cases calculated afferent barrage is less when SFL is present (Djouhri et al., 2006). We therefore speculate that SFL is only manifest when C fibre afferent input to the spinal cord reaches a certain level, and that when it is less than this, more subtle hindpaw postural changes such as ventroflexion may occur. There has been debate about whether ventroflexion is representative of motor damage (Na et al., 1996, Kissin et al., 2007), as it is most frequently described following injury of a mixed nerve (Attal et al., 1990). Our findings support the conclusion ventroflexion is a consequence of sensory rather than motor damage, as the saphenous nerve contains no motor fibres.

In contrast to studies on traumatic nerve injury, implicating changes in A fibre properties in the development of ongoing pain and mechanical allodynia in both experimental models (Kajander and Bennett, 1992, Liu et al., 2000, Ma et al., 2003) and humans (Campbell et al., 1988), we found no evidence for alteration of the properties of intact A fibres in PSNI. In the majority of studies in which A fibres show ongoing activity, the afferents are disconnected from the peripheral terminal, either by complete axotomy, or by tight ligation (Kajander and Bennett, 1992, Bongenhielm and Robinson, 1998, Tal et al., 1999, Pitcher and Henry, 2000, Pitcher and Henry, 2004). Recent reports show however that A fibres have reduced mechanical thresholds following regeneration (Jankowski et al., 2009), and develop ongoing activity in inflammation (Xiao and Bennett, 2007), and on more subtle neuropathy in which fibre degeneration does not occur (Xiao and Bennett, 2008).

The behavioural mechanical allodynia seen in the PSNI model could be attributable to sensitisation of spinal cord neurones, due to the increased ongoing activity in mechanoreceptive afferents (Schwartzman et al., 2001). To determine whether peripheral sensitisation might also contribute to the behavioural changes, we examined mechanical thresholds in the afferents. We found no evidence to suggest that mechanical thresholds were reduced in intact A or C fibre afferents after PSNI. The neuropathy induced by the AIDS drug ddC (2^′^,3^′^-dideoxycytidine) (Chen and Levine, 2007) was very similar to that seen here in PSNI. In the ddC model allodynia develops rapidly (Joseph et al., 2004), and is associated with C fibre conduction velocity slowing, but no alteration in C fibre mechanical threshold (Chen and Levine, 2007). In contrast ethanol-induced neuropathy was associated with reduced mechanical thresholds (Chen and Levine, 2007). Mechanical thresholds are unchanged (Ali et al., 1999, Ji et al., 2007) or reduced (Shim et al., 2005) following spinal nerve ligation. The trend for reduced mechanical thresholds at day 7 in our study might indicate a reduction of threshold in a subpopulation of afferents, as reported in inflammation (Dunham et al., 2008). As mechanical thresholds were not reduced in PSNI, we conclude that peripheral sensitisation does not contribute to the mechanical allodynia. At day 7, however, while total afferent barrage is less than that seen on day 3 (∼1.7 KHz versus 0.7 KHz), animals show equivalent mechanical allodynia to that seen at day 3. This is consistent with the hypothesis that continued behavioural allodynia in PSNI may require continued afferent barrage, albeit at a lower level than that required to induce SFL (Pitcher and Henry, 2008). Supporting the conclusion that C, rather than A fibre input is fundamental to the generation of behavioural mechanical allodynia are reports that ablation of TRPV1-expressing C fibre afferents reduces these behaviours following traumatic peripheral nerve injury (Jang et al., 2007, Kim et al., 2008).

Although cold allodynia is a frequent finding following nerve damage in humans (Jorum et al., 2003), the mechanisms of cold allodynia following nerve injury are poorly understood. In contrast to the mechanical allodynia seen in PSNI, the cold allodynia continued to increase in magnitude up to day 14. The increased cold allodynia over the first 7 days paralleled the increase in afferent input from cooling sensitive fibres, and in cooling-evoked activity in cooling-sensitive afferents, despite a decrease in the numbers of active afferents at 3 days. This is in contrast to previous findings (Ji et al., 2007), where all observed changes were seen in intact Aδ fibres and C fibres did not contribute to cold allodynia. We do not know whether the mechano-cool afferents that we studied also express TRPV1, but it is possible that these afferents with increased ongoing and cooling-evoked activity may represent the group of TRPV1-expressing afferents implicated in behavioural cold allodynia (Tanimoto-Mori et al., 2008).

Alteration in skin temperature would be expected to evoke activity in cooling-sensitive afferents. Inconsistent changes in skin temperature in clinical neuropathic conditions have been reported (e.g. increased (Gratt et al., 1995) or decreased (Kang et al., 2003)) even in the same condition (e.g. diabetic neuropathy (Boyko et al., 2001, Nabuurs-Franssen et al., 2002)). In experimental models, altered cutaneous blood flow and temperature are consequences of damage to sympathetic efferents (Hord et al., 1999). The sympathetic component in the saphenous nerve is similar to that in the sciatic (Baron et al., 1988). The effect of PSNI on skin temperature might be expected to be equivalent to sciatic damage which results in acute changes in skin temperature (no change up to 1 week, ∼1 °C increase at 1–2 weeks). After 2 weeks, skin temperature is significantly reduced (Sotgiu et al., 1995, Hord et al., 1999). Both cooling evoked and ongoing activity were significantly increased in CMC afferents, suggesting that sensitisation of CMC afferents, rather than changes in skin temperature underlie the short-term changes in the properties of the CMC afferents in this study.

Our data suggest that the increased ongoing discharge in CMC units in the saphenous nerve could lead to central sensitisation, contributing to the secondary cold hyperalgesia exhibited on the plantar surface of the foot. Cooling allodynia in humans is reported to be both ipsilateral and contralateral to an injury (Baron and Maier, 1995, Jorum et al., 2003) and in experimental animals and humans, cold allodynia can be blocked by intrathecal NMDA receptor blockade (Burton et al., 1999, Jorum et al., 2003). These observations suggest that central sensitisation plays an important role in cold allodynia, although there are few published studies investigating the spinal mechanisms of sensitisation to peripheral cool/cold stimulation, particularly after peripheral nerve injury. Although cold-specific neurones have been described in lamina I of the spinal cord (Christensen and Perl, 1970, Dostrovsky and Craig, 1996), these neurones receive input from the “classical” cooling-responsive units not included in our study. It is therefore most likely that the afferents we studied activated wide dynamic range (Class 2) (Menetrey et al., 1977) spinal neurones that also receive mechanical input. In the only study to date on cold-evoked responses in WDR neurones after nerve injury, these spinal neurones were shown to exhibit enhanced responses to cold stimulation (Brignell et al., 2008) suggestive of central sensitisation. It is possible that our observations on the changed properties of primary afferents, leading to significant barrage, might explain this central sensitisation to cold in nerve injury.

In this study we describe primary afferent changes consequent to peripheral nerve injury, including ongoing activity and increased cooling-evoked responses. These altered properties that may contribute to altered mechanical and cooling behaviours by induction and maintenance of central sensitisation.
